# How to Grow a Lung: Applying Principles of Developmental Biology to Generate Lung Lineages from Human Pluripotent Stem Cells

**DOI:** 10.1007/s40139-016-0102-x

**Published:** 2016-04-18

**Authors:** Briana R. Dye, Alyssa J. Miller, Jason R. Spence

**Affiliations:** Department of Cell and Developmental Biology, University of Michigan Medical School, Ann Arbor, Michigan 48109 USA; Department of Internal Medicine, University of Michigan Medical School, Ann Arbor, Michigan 48109 USA; Department of Cell and Molecular Biology, University of Michigan Medical School, Ann Arbor, Michigan 48109 USA; Center for Organogenesis, University of Michigan Medical School, Ann Arbor, Michigan 48109 USA

**Keywords:** Directed differentiation, Human pluripotent stem cell, Organoid, Lung development, Airway, Alveoli

## Abstract

The number and severity of diseases affecting human lung development and adult respiratory function has stimulated great interest in new in vitro models to study the human lung. This review summarizes the most recent breakthroughs deriving lung lineages in a dish by directing the differentiation of human pluripotent stem cells. A variety of culturing platforms have been developed, including two-dimensional and three-dimensional (organoid) culture platforms, to derive specific cell types and structures of the lung. These stem cell-derived lung models will further our understanding of human lung development, disease, and regeneration.

## Introduction

**Fig. 1:**
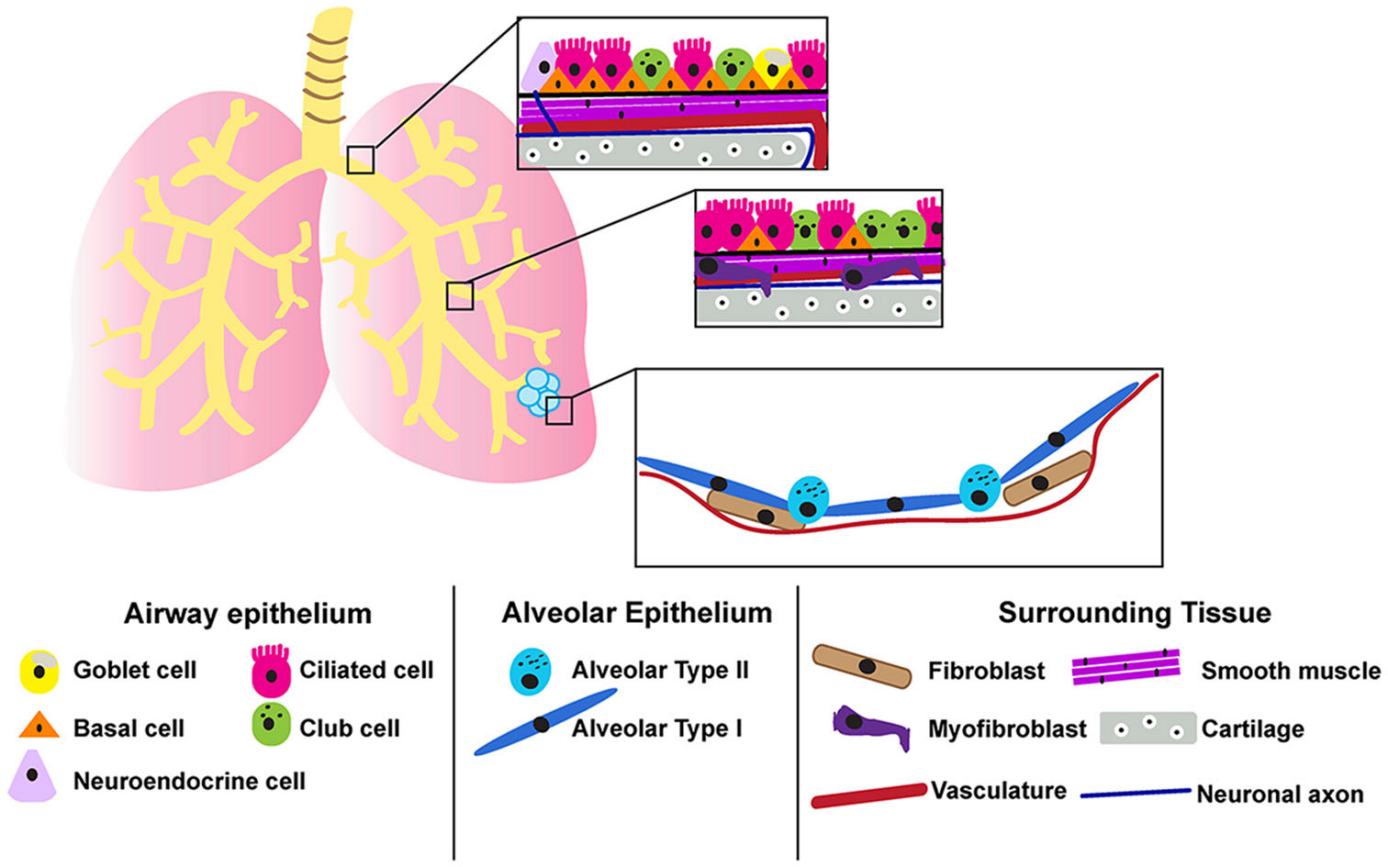
A diagram summarizing the lung cell types in and surrounding the airways and alveoli. Upper airways are surrounded by cartilage (*brown*) and smooth muscle (*pink*). The upper airway epithelium is lined with basal cells (*orange*) with ciliated (*pink*), goblet (yellow), club (green), and neuroendocrine (*light purple*) cells adjacent to the basal cells facing toward the lumen of the airway. The lower airways possess less basal cells and consist of mostly ciliated and club cells with surrounding tissues consisting of smooth muscle, myofibroblasts (*purple*), and patches of cartilage. Vessels (*red line*) and neurons (*blue line*) line both the upper and lower airways. The alveolar sacs consist of elongated AECI (*light blue*) and cuboidal AECII cells (*dark blue*) that are lined with thin vessels in order for gas exchange to occur with few fibroblasts scattered outside the alveolar sac. Note that only the most abundant lung cell types are depicted (Color figure online)

The lungs are the main organ of the respiratory system and function to exchange gas, taking in oxygen and expelling carbon dioxide. The lungs are organized in a branched, tree-like structure that has two major anatomical features, the conducting airways and alveoli. The conducting airways are arranged in a series of tubes that become progressively smaller as they move gas into the alveoli. The conducting airways start as a singular tube, the trachea, which splits into two main bronchi. The main bronchi then further split and branch into smaller airway tubes called the conducting bronchioles. The walls of the conducting airways consist of epithelial cell types surrounded by supporting mesenchyme including fibroblasts, smooth muscle, cartilage, vasculature, and neurons extending the length of the airways (Fig. 1). Just before the bronchioles terminate they form a small tube called the alveolar duct, which leads into the thin sac of cells that make up the alveoli. The alveolar epithelium is tightly associated with a capillary network in order for efficient gas exchange to take place.

In order to accommodate diverse functions, the human lung possesses several specialized cell types. The conducting airways are predominated by three epithelial cell types: ciliated, goblet, and basal cells, with ciliated cells being the most dominant cell type in human airways [1]. Ciliated cells have multiple cilia that beat in a synchronous rhythm in order to maintain the flow of mucus across the airway epithelium [2, 3]. Goblet cells are secretory cells that secrete mucus on the surface of the airway [4]. The basal cells are the adult stem cells of the airways [5–9]. In humans, basal cells line the main bronchi and large bronchioles, but begin to decrease in number in the smaller bronchioles toward the alveoli [1, 8, 10, 11]. Other, sparse populations of cells in the airway include club cells, which are secretory cells that secrete lubricating glycosaminoglycans and antimicrobial peptides into airways [12], and pulmonary neuroendocrine cells which are innervated on the basal surface and store proteins that are released under a physiological stimulus such as hypoxia [13–16]. The alveolar epithelium is composed of two epithelial cell types, alveolar epithelial type I cells (AECI) and alveolar epithelial type II cells (AECII) (Fig. 1). AECIs form a thin, squamous epithelium that covers the majority of the alveolar surface and exchanges gas with neighboring capillaries through diffusion [17–20]. AECIIs have a cuboidal shape and secrete surfactant proteins to reduce the surface tension of the alveolar sacs, allowing them to expand and contract without collapsing as breathing takes place [18, 20–22].

The complex architecture of the adult lung is established during early embryonic development. The epithelium of the lung is derived from the embryonic endoderm while much of the structural support of the lung, including fibroblasts, smooth muscle, and cartilage, is derived from the embryonic mesoderm. Lung development begins as two buds forming off of a tube of endoderm surrounded by mesoderm called the gut tube. The gut tube gives rise to the entire gastrointestinal tract and associated organs, including lungs, thyroid, liver, and pancreas [23–26]. The primary lung buds emerge from the gut tube, invade the surrounding mesoderm and continue to elongate in tight association with the mesoderm throughout development. The lung buds then undergo branching morphogenesis, in which epithelial bud tips continuously bifurcate, forming new branches, until a stereotyped tree-like pattern is formed [27]. During branching, a proximal–distal lung axis is established, with proximal lung epithelium giving rise to conducting airways, and the distal tips eventually terminally differentiating into the alveolar sacs (for more detailed reviews see: [28, 29]).

Since lung development is so critical for neonatal life, it has been the focus of intense study. In addition, lung diseases are prevalent and can be caused by environmental exposure to pathogens or damaging toxins; however, in many cases, chronic lung diseases are often a result of both genetics and environment. Much of our understanding of aberrant lung development and adult disease has resulted from using animal models and cell culture systems. These approaches have yielded a tremendous amount of insight into the mechanisms that cause disease; however, there are many aspects of human biology and disease that are not reflected in animal models or cell culture systems. In order to address the need for physiologically relevant human model systems, the past decade has seen several fields turn to human pluripotent stems cells (hPSCs), which include both embryonic stem cells and induced pluripotent stem cells, in an attempt to generate specific cell types and even complex organ-like tissue in vitro [30••, 31–34]. Indeed, the lung field has seen rapid growth in the number of exciting reports generating lung tissue in vitro [35•, 36•, 37•, 38•, 39•, 40•, 41•, 42•, 43•, 44•, 45•]. In this review article, we will introduce the developmental biology framework that forms the basis used by many groups attempting to differentiate lung tissue from hPSCs. We will then highlight successful efforts to generate ‘lung tissue in a dish,’ and discuss the implementation of these varied human model systems, followed by a discussion of their strengths and limitations.

## Endoderm and Anterior Foregut Specification

hPSC-derived tissues are often generated using directed differentiation, a process that aims to recapitulate the signals that drive cell or organ-specific differentiation in the embryo in an in vitro environment [36•, 37•, 38•, 39•, 40•, 41•, 42•, 43•, 44•, 45•, 46•]. In the case of the lung, this entails a series of sequential steps to derive endoderm, then anterior foregut endoderm and induction of lung progenitor cells, followed by lung-specific cell type differentiation and maturation. It has long been appreciated that Nodal signaling is necessary to form definitive endoderm during gastrulation in animal models, and is also sufficient to convert non-endodermal lineages (ectoderm) into endoderm in early embryonic tissue [26, 46–50]. In cell culture, ActivinA, which is a Tgfβ superfamily member, stimulates the same signaling pathways as Nodal and has been widely used to successfully induce definitive endoderm differentiation from hPSCs [30••, 34, 39•, 51].

Following gastrulation, the embryo undergoes a series of morphological movements that give rise to the gut tube, which is an endodermal tube surrounded by mesoderm. Embryo and gut tube patterning are guided by a multitude of secreted proteins that both stimulate and inhibit different signaling pathways in order to establish the domains of the gut tube, referred to as the foregut, midgut, and hindgut which form along the anterior-posterior axis of the embryo (for more detailed reviews, see: [26, 52]).

Several secreted inhibitors help to establish the anterior identity of the foregut endoderm, which will give rise to the lungs and thyroid. As the endoderm migrates to form the gut tube during gastrulation, cells at the anterior pole of the embryo encounter secreted Nodal inhibitors Lefty1 and Cerberus-like (Cerl) and BMP inhibitors Chordin and Noggin, which inhibit posterior patterning [53–55]. In Xenopus laevis, the Wnt antagonist Sfrp5 is secreted from the endoderm to maintain foregut identity [56]. In line with embryonic regulation of anterior endoderm patterning, an activator/inhibitor screen conducted in hPSC-derived endoderm that included inhibitors and activators of Nodal, Hedgehog (HH), WNT, RA, BMP, and TGFβ signaling pathways identified that inhibition of the BMP and TGFβ signaling pathways robustly induced anterior foregut genes [57••]. The observation that inhibition of BMP and TGFβ (“dual smad inhibition”) potently stimulates a foregut endoderm fate has been widely adopted by the lung differentiation field [38•, 39•, 40•, 41•, 45•, 57••]. Interestingly, although secreted Wnt inhibitors help maintain foregut identity in vivo, inhibition of Wnt signaling is not necessary to induce foregut endoderm in vitro [36•, 37•, 39•, 40•, 41•, 45•, 57••].

## Morphogenesis in a Dish

In hPSC-derived endoderm cultures, it has been shown that stimulating WNT and FGF signaling pathways (via WNT3A and FGF4) cause self-aggregation of three-dimensional cell clusters, called spheroids, that delaminate from the tissue culture monolayer [34, 39•, 51, 58]. While the mechanisms driving three-dimensional spheroid formation in endoderm cultures is not known, it is interesting to speculate that FGF4 and WNT are acting, as they do in vivo, to drive cellular reorganization and migration [59–63].

In addition to driving three-dimensional morphogenesis in a dish, WNT3A and FGF4 are potent stimulators of the CDX2+ intestinal lineage in vitro [34]. However, simultaneous stimulation of WNT/FGF signaling in combination with the in vitro anterior foregut promoting conditions (dual smad inhibition), caused spheroids to take on an anterior foregut fate, including the expression of, NKX2.1, PAX8, and SOX2 [39•]. Taken together, these studies demonstrated that hPSC-derived definitive endoderm can be directed to become anterior foregut endoderm by mimicking in vivo patterning signals.

## Lung Induction

During early development, the lung primordium forms along the ventral anterior foregut and expresses the transcription factor Nkx2.1, which is required for lung fate [64, 65]. Extensive study of lung specification has identified several signaling pathways that are critical for this process, including Fgf, Bmp, Wnt, Hedgehog (Hh), and RA signaling pathways (reviewed in [28, 66]). Bmp signaling to the anterior-ventral foregut endoderm is important to prime the lung domain by inhibiting Sox2, allowing lung specification to take place [66, 67]. Additionally, Fgf2 secreted from the cardiac mesoderm, which sits adjacent to the ventral foregut, and Fgf10 secreted from the surrounding lung mesoderm are necessary for Nkx2.1 expression and lung formation [68–71]. Similarly, Wnt ligands signaling from the lung mesoderm to the ventral foregut endoderm are also necessary for Nkx2.1 expression and lung induction [72–75]. RA promotes Nkx2.1 expression and lung bud formation in part by inhibiting TGFβ and the Wnt antagonist Dkk1, allowing Fgf and Wnt signaling to occur [76, 77]. Hh signaling is also critical for lung development, as concurrent deletion of the Hh signaling transcription effectors Gli2 and Gli3 leads to lung agenesis [78].

**Fig. 2:**
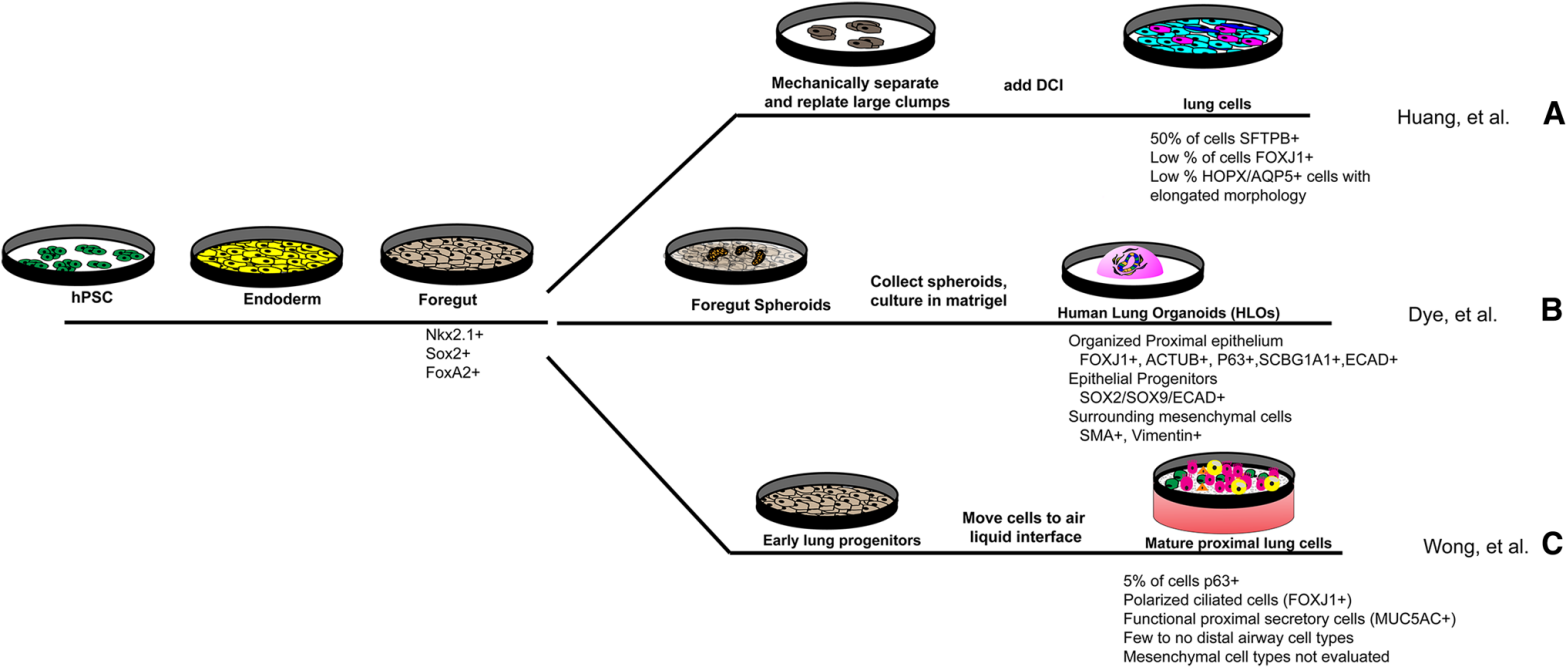
The majority of protocols to derive lung cell types from hPSCs have taken a directed differentiation approach. hPSCs are first treated with growth factors, including ActivinA, to derive endoderm, which is further treated to become anterior foregut endoderm. Anterior foregut endoderm cells are marked by Nkx2.1, FoxA2, and Sox2 transcription factors. Different groups have used various methods to derive lung cell types after this stage. **a** Huang et al. grew foregut cultures for 15 days, after which cells were broken up and remaining large clumps were re-plated. On day 25, cells were treated with a DCI cocktail, which has been shown to induce alveolar specific cell-type gene expression in vitro [34, 94]. After 48 days in monolayer culture, the majority of cells expressed the AECII marker, SFTPB. A low number of cells also expressed the proximal ciliated cell marker FOXJ1, and some cells expressed markers for mature AECI (HOPX, AQP5) cells and exhibited elongated nuclei. **b** Dye et al. treated foregut cultures with FGF4 and Wnt to induce three-dimensional foregut spheroids, which were cultured in a matrigel droplet. These lung organoids persisted in culture for over 100 days and contained an organized epithelium containing cells positive for proximal airway markers FOXJ1/ACTUB (ciliated cells), p63 (basal stem cells), SCBGA1A1 (club cells), and surrounding mesenchymal tissue positive for smooth muscle (SMA) and vimentin (VIM). Additionally, organoids contained some cells that stained positive for AECI and AECII cell makers, but organized alveolar structures were not observed. **c** Wong et al. used multiple growth factor cocktails over the course of 35 days to induce lung progenitor cells in a monolayer culture. After roughly 5 weeks in culture, cells were moved to an air–liquid interface environment, which resulted in maturation of lung cells. The majority of cells expressed mature proximal cell markers for ciliated cells (FOXJ1) or goblet cells (MUC5AC), and cells exhibited distinct polarization, with the basal side of cells facing the media and the apical side of cells facing the air, similar to the in vivo polarization of lung cells with apical surfaces facing the airway lumen

Since the events required for lung induction are complex and involve multiple signaling pathways that are controlled in very tight temporal and spatial manner in vivo, translating these developmental paradigms in a dish has proven to be challenging. However, many groups have generated hPSC-derived NKX2.1+ lung progenitor populations with varying levels of efficiency (Fig. 2a–c) [35•, 36•, 37•, 38•, 39•, 40•, 41•, 43•, 44•, 45•]. The majority of methods to derive lung tissue in vitro have treated endoderm, or anterior foregut endoderm, in monolayer cultures with FGFs, BMPs, WNTs, and RA to induce NKX2.1+ endoderm. Approaches to induce three-dimensional lung organoids have used a combination of factors to promote spheroid formation by activating FGF4 and WNT signaling, while simultaneously specifying foregut with dual smad inhibition and inducing lung.

To date, the field has primarily focused on differentiating lung epithelial cells from hPSCs, however, the lung mesenchyme also plays a critical role in sending and receiving signals and physically interacting with the lung epithelium during early development [28]. Given the important contribution of the mesenchyme to lung development and lung function, modeling this aspect of lung development in vitro represents a significant opportunity for furthering the field.

## Proximal–Distal Patterning in the Lung

Following the induction of an Nkx2.1+ field of lung progenitor cells, the primordial lung buds undergo branching morphogenesis, giving rise to a patterned epithelium that consists of Sox2+ proximal airways and Sox9+/Id2+/Nmyc+ actively branching epithelium that will eventually differentiate, giving rise to the alveoli [79–82]. Diffusible growth factors including Bmp4, Fgfs, and Wnts are essential for establishing and maintaining the proximal–distal pattern of the lung. Bmp4 and Wnt2/2b expression in the distal mesenchyme and Wnt7a originating in the distal epithelium signal to the distal epithelium and are essential for maintaining the distal epithelium and promoting branching [83–87]. In mice, blocking Wnt signaling by conditional loss of β-Catenin from the epithelium results in disrupted branching and expansion of the proximal epithelium [84, 88]. Similarly, Fgf7 and Fgf10 signaling from the distal mesenchyme to the epithelium promotes epithelial growth and branching, and Fgf10 plays a role in maintaining distal progenitor cells during branching morphogenesis [89–93]. RA promotes branching by increasing Fgf10 expression in the lung mesenchyme [94]. Once the branching program is complete, the distal epithelium differentiates into the alveolar cell types, AECI and AECII. In the mouse lung, both of these cell types arise from a distal bipotent progenitor [95], however; the factors that regulate cell fate choice and differentiation of alveolar cell types in the embryo are not well understood.

Efforts to derive distal and alveolar cell types in vitro have attempted to recapitulate the signaling factors active around distal buds during branching in the developing embryo. To date, Huang et al. have discerned the most efficient methods to generate AECI and AECII cells from hPSCs (Fig. 2a) [38•]. hPSC-derived foregut endoderm was cultured with Chir99021 (a GSK3β antagonist that stabilizes β-catenin), BMP4, FGF10, FGF7, and RA for 15 days at which time the cells were cultured with Chir99021, FGF10, and FGF7 for an additional 25 days, followed by treatment with dexamethasone, cAMP, and isobutylmethylxanthine (DCI), which stimulates alveolar cell-specific gene expression in vitro [35•, 96]. Interestingly, dexamethasone is administrated to premature infants to accelerate fetal lung maturation, which results in enhanced surfactant secretion from AECIIs [97, 98]. These sequential steps resulted in cells expressing AECII protein SFTPB in over 50 % of all cells in the culture, with some SFTPB+ cells displaying lamellar bodies and functional release and uptake of surfactant protein. These factors also induced cells to express AECI cell-specific markers. While these cells displayed the flat, elongated nuclei typical of AECI cells, they did not exhibit elongated cell bodies or form multi-cellular sac-like alveolar structures [38•]. Others have found that seeding NKX2.1+ lung progenitors onto human lung extracellular matrix (ECM) proteins significantly enhanced AECI and AECII cell differentiation, indicating that both the physical and chemical environments are important for alveolar differentiation in vitro [42•].

More recently, attempts have been made to differentiate three-dimensional lung-like tissues in vitro by recreating the embryonic distal lung environment in a dish (Fig. 2b) [39•]. Three-dimensional foregut spheroid cultures grown in an extracellular matrix (Matrigel) with high concentrations of FGF10 led to the formation of lung organoids. Lung organoids possessed a distal alveolar cell population that expressed bipotent progenitor markers including SFTPC, HOPX, and SOX9 [95] with a few cells expressing mature AECI and AECII markers, PDPN and SFTPB, respectively. Alveolar cell types were found in distinct regions of the lung organoid, but true alveolar sac-like structures were not observed [39•].

Thus, one of the hurdles that remain in hPSC-derived lung differentiation is to successfully recapitulate alveolar structure in vitro. Alveolar-like structures have been derived from primary human AECII cells co-cultured with fetal human lung fibroblast cell lines forming alveolar spheres (alveolospheres). The cultures consist of phenotypic AECI and AECII cells demonstrating proof of concept that achieving such three-dimensional alveolar structure is possible [99]. Similar approaches have been attempted with hPSC-derived tissues, where the cell surface marker carboxypeptidase M (CPM) was used to enrich NKX2.1+ progenitors using fluorescent-activated cell sorting (FACS). CPM-purified lung progenitors were cultured in a three-dimensional extracellular matrix along with FGF7, DCI, and a fetal human lung fibroblast cell line. This resulted in three-dimensional spheres composed of cells expressing AECI and AECII markers, yet proper AECI morphology was not demonstrated [41•]. Although effective, one of the drawbacks to this approach is that it is unclear if the human fetal feeder cells are providing a physical niche through cell–cell contact, and/or if they secrete important factors [41•, 99]. While several groups have shown success obtaining distal epithelial cell types in vitro, additional work is needed to better understand the mechanisms that control AECI and AECII differentiation to improve upon the efficiency of the differentiation and deriving alveolar structure.

## Proximal Airway Differentiation

In the developing embryo, a variety of factors are important for promoting proximal airway fate in mouse lungs [100]. As development progresses, the Sox9/Id2/Nmyc bud tips differentiate into proximal tissue and express the marker Sox2. The multipotent Sox2+ airway population will give rise to neuroendocrine, club, ciliated, goblet, and basal cells [28]. Activation of Notch signaling promotes Sox2+ cells to differentiate into secretory cells, whereas inhibition of Notch signaling promotes ciliated cells and neuroendocrine cell fates [101–103]. During late lung development and adult homeostasis, the Fgf signaling ligand Fgf18 is necessary for maintaining basal stem cells in the proximal airways, and Notch signaling acts to control the balance of secretory and ciliated cell types [5, 9, 100, 104, 105].

In order to differentiate proximal airway cell types from hPSC-derived endoderm, approaches to both promote proximal airway differentiation and to reduce distal airway differentiation have been taken (Fig. 2c). In order to steer cells away from a distal fate, BMP4 concentrations were reduced and FGF18 was added to promote proximal cell fates, an approach that resulted in over 50 % of the cells expressing the basal stem cell marker P63. To further mature proximal cell types, cultures were moved to an air–liquid interface (ALI) resulting in an increase in mature airway cells and polarization of the airway epithelium. The cells on the ALI expressed markers for goblet, club, and ciliated cells along with basal cells [36•]. Together, these results suggest that foregut endoderm can be directed to a proximal lung fate by a combination of reducing factors that promoted distal fate and adding factors that promoted proximal fate. However, the role of the polarizing ALI environment and the undefined growth factors present in commercially purchased ALI media in this approach leaves room for further exploration.

In addition to the ALI platform, three-dimensional airway cultures have been generated from hPSCs, applying similar concepts used to derive “bronchosphere” from human and mouse primary basal cells, an approach first establishing that human basal cells can self-renew and generate ciliated and club cells in vitro [5]. Successful growth of bronchospheres from hPSCs has been achieved using purified CPM+ foregut progenitor cells, similar to the approach used for alveolarspheres [40•, 41•]. Using commercially available ALI media and Notch inhibition, the sorted CPM+ population formed spheres in a three-dimensional matrix from single cells. Spheres consisted of mostly ciliated cells interspersed with neuroendocrine cells with even fewer basal cells and secretory cells. These hPSC-derived bronchospheres represent the first report of beating ciliated cells that are not derived from a primary cell line; however the cells do not organize into a pseudostratified epithelium, as is the case with the lung in vivo [40•]. Foregut spheroids grown in a three-dimensional ECM, Matrigel, overlaid with media containing high concentrations of FGF10 also gave rise to proximal airway-like structures that formed a polarized epithelium organized in a cyst containing a lumen, and surrounded by mesenchyme (Fig. 2b). The epithelium consisted of basal cells close to the mesenchymal tissue, with the adjacent epithelial cell types facing toward the lumen and expressing an early marker of ciliated cells, FOXJ1 [39]. High FGF10 was required for airway-like epithelium to differentiate in lung organoids, and may act to maintain the basal cell population in lung organoids, however, this has not yet been formally tested. Consistent with high FGF10 promoting proximal fates, recent evidence in mice suggests that high levels of ectopic Fgf10 expressed during embryonic development can increase the number of P63+ basal stem cells present in the lung [90]. Interestingly, as has been demonstrated in both lung organoids and other hPSC-derived tissues, including hepatocyte-like, β-cell-like, intestinal, stomach, and cerebral organoids, even in long-term cultures, the organoids retain a transcriptional profile similar to fetal tissue than to adult tissue [33, 39•, 51, 106–108]. Thus, an important unresolved problem in the field is how to overcome this developmental barrier in vitro in order to obtain more mature adult-like tissue. Achieving this goal could require modulation of the physical or chemical environment to induce further maturation of the tissue.

## Future Directions: Next Steps for In Vitro Lung Models

### Branching Morphogenesis

Although the field has achieved success in deriving both proximal and distal cell types from hPSCs in two-dimensional and three-dimensional cultures [35•, 36•, 37•, 38•, 39•, 40•, 41•, 42•, 43•, 44•], to date, there have been no reports of in vitro human systems that mimic the complex three-dimensional morphology of epithelial branching structures seen in the embryo and *bona fide* alveolar structures have not been described. Therefore, the development of an hPSC-derived in vitro model of lung branching will be hugely beneficial for furthering our understanding of human lung development and associated pathologies. The development of such a model would provide a critical tool for investigating the complex interactions between the ECM, force gradients, diffusible morphogens, and specific genetic programs active during the development, disease, and repair of the human distal lung regions.

Much of the work in the branching morphogenesis field has focused on cell signaling and genetic factors that regulate this process. However, the physical environment also plays a role in directing branching. It is well established that cues from the ECM, as well as inputs from vascular endothelial cells and neurons play important roles in directing this process. As distal lung buds begin to bifurcate, a thick deposit of ECM is present at the bud cleft, while Matrix Metalloprotinases (MMPs) appear to play a role in degrading the ECM around growing bud tips [109]. Recent work has shown that mouse lung buds isolated from the mesenchyme can be cultured in three-dimensional ECM environments of increasing stiffness and as stiffness increases, the buds form more branches, suggesting the mechanical force of the ECM can play a role in directing branching [110]. In addition to mechanical influences, vascular endothelial cells play a role in early branching morphogenesis and in maintaining lung epithelial stem cells in vivo [99, 111–114]. Similarly, migrating neural crest cells will invade the early lung and become neurons, which are necessary for proper branching morphogenesis [115–117]. Together, this evidence from animal models suggests that mechanical forces, as well as inputs from vascular endothelial cells and neuronal innervation all play essential roles in defining and maintaining the lung architecture during lung branching. Yet, so far, these critical physical regulators of lung organogenesis have eluded translation into hPSC-derived in vitro models. Careful attention to defining an optimal physical environment and cues from closely associated non-lung cell types may improve the complex architecture and patterning of in vitro hPSC-derived lung tissue cultures in the future, and will be a critical next step to advancing models of human lung development, disease, and regeneration.

In addition to improving our understanding of the physical environment of the developing lung, detailed investigation into the types, concentrations, and gradients of diffusible morphogen signals that promote a distal versus proximal lung fate, and tissue engineering approaches to appropriately deliver regulated morphogen gradients, may also improve existing techniques for generating hPSC-derived models of the human lung.

### Functional Testing

Although many cell markers exist to identify specific lung cell types derived from hPSCs, the functional studies implemented thus far have been limited to surfactant protein production and uptake for AECII cells [38] and recording ciliated cell movement [40•]. These tests show a very specific function for limited cell types in the lungs. Unfortunately, there is no comprehensive functional test for in vitro lung cultures. In rats, decellularized lung matrices have been seeded with neonatal rat lung epithelial plus vasculature and were functionally assessed for their ability to sustain life for a short period of time [118, 119]. In addition, human decellularized lung matrices have been hooked up to a bioreactor to create an environment for fluid and air exchange to take place [118, 120–122]. However, it is unclear if reseeding an entire adult human lung matrix is feasible for such bioreactor studies, and either more efficient methods for reseeding need to be developed, or more realistic lung structures with branched tubular networks need to be differentiated *de novo.*

Along with testing overall organ function, cell-specific tests will also be necessary to test lung cellular function. Cellular tests for AECII and ciliated cells have been demonstrated [38•, 40•], but there is still no functional test for AECI or secretory cells. AECI cells function mainly to exchange gas; therefore, in vitro methods to measure gas diffusion in a highly sensitive fashion may need to be developed. For secretory cells, sensitive methods to stimulate and measure proteins released by secretory cells (goblet, club cells) will also be highly valuable for establishing function. Measuring protein secretion of single cells in vitro has been established, but ideally the goal would be to measure protein secretion within three-dimensional airway structures [123, 124]. Taken together, two types of functional tests need to be implemented: the first to test the overall lung function of gas exchange and airway movement and the second to hone in on a specific cellular function.

## Conclusions

Currently, the majority of hPSC-derived human lung models are generated by treating hPSCs with a series of growth factor combinations that attempt to mimic the signaling milieu observed in vivo to direct differentiation to lung-specific cell types. These methods have resulted in a diverse range of model systems and this approach has been effective in generating many lung cell types and relevant lung architecture. Yet, gold standard functional tests need to be established to validate the diverse findings using different methodologies. These tests will ideally include both cellular function and assessment of tissue/organ-level function. In just the past few years, various human lung models have been derived that can be applied to study lung development, disease, and tissue regeneration. However, mimicking the in vivo environment of the lung by including other tissues including mesenchyme, vasculature, and neurons will make for an even more realistic human model moving forward.
